# Effects of Curcumin in a Mouse Model of Very High Fat Diet-Induced Obesity

**DOI:** 10.3390/biom10101368

**Published:** 2020-09-25

**Authors:** Iurii Koboziev, Shane Scoggin, Xiaoxia Gong, Parvin Mirzaei, Masoud Zabet-Moghaddam, Mohammad Yosofvand, Hanna Moussa, Yava Jones-Hall, Naima Moustaid-Moussa

**Affiliations:** 1 Department of Nutritional Sciences, Texas Tech University, College of Human Sciences, Lubbock, TX 79409-1270, USA; shane.scoggin@ttu.edu (S.S.); naima.moustaid-moussa@ttu.edu (N.M.-M.); 2 Obesity Research Institute, Texas Tech University, College of Human Sciences, Lubbock, TX 79409-1270, USA; masoud.zabet@ttu.edu (M.Z.-M.); hanna.moussa@ttu.edu (H.M.); 3 Center for Biotechnology and Genomics, Texas Tech University, Canton & Main Experimental Sciences Building, Lubbock, TX 79409-3132, USA; xiaoxia.gong@ttu.edu (X.G.); parvin.mirzaei@ttu.edu (P.M.); 4 Department of Mechanical Engineering, Texas Tech University, 100 Engineering Center Box 43103, Lubbock, TX 79409-3103, USA; mohammad.yosofvand@ttu.edu; 5 Department of Comparative Pathobiology, Purdue University, 725 Harrison St, West Lafayette, IN 47907, USA; yavajh@cvm.tamu.edu

**Keywords:** mouse models, very high fat diet, obesity, osteoarthritis, chronic inflammation, curcumin

## Abstract

Worldwide rates of Western-diet-induced obesity epidemics are growing dramatically. Being linked with numerous comorbidities and complications, including cardiovascular disease, type 2 diabetes, cancer, chronic inflammation, and osteoarthritis (OA), obesity represents one of the most threatening challenges for modern healthcare. Mouse models are an invaluable tool for investigating the effects of diets and their bioactive components against high fat diet (HFD)-induced obesity and its comorbidities. During recent years, very high fat diets (VHFDs), providing 58–60% kcal fat, have become a popular alternative to more traditional HFDs, providing 40–45% total kcal fat, due to the faster induction of obesity and stronger metabolic responses. This project aims to investigate if the 60% fat VHFD is suitable to evaluate the protective effects of curcumin in diet-induced obesity and osteoarthritis. B6 male mice, prone to diet-induced metabolic dysfunction, were supplemented with VHFD without or with curcumin for 13 weeks. Under these experimental conditions, feeding mice a VHFD for 13 weeks did not result in expected robust manifestations of the targeted pathophysiologic conditions. Supplementing the diet with curcumin, in turn, protected the animals against obesity without significant changes in white adipocyte size, glucose clearance, and knee cartilage integrity. Additional research is needed to optimize diet composition, curcumin dosage, and duration of dietary interventions to establish the VHFD-induced obesity for evaluating the effects of curcumin on metabolic dysfunctions related to obesity and osteoarthritis.

## 1. Introduction

The global epidemic of obesity represents one of the most urgent healthcare challenges worldwide. Several strategies were tested to reduce obesity and alleviate its associated metabolic and inflammatory diseases including osteoarthritis. Among these, novel therapeutic approaches based on enriching the diet with bioactive foods have gained attention of nutritionists and biomedical researchers. Low-molecular-weight plant polyphenol curcumin (diferuloymethane), obtained from turmeric (*Curcuma longa*) roots, has been used in medicine and as a spice in the countries of South-East Asia for 4000 years [1]. The beneficial effects of curcumin against obesity, metabolic disorders, inflammation, malignancies, and a variety of other pathophysiologic conditions have been ascertained in numerous clinical trials [2]. The anti-inflammatory effects of curcumin relate to its ability to modulate intracellular signaling via the nuclear factor kappa-light-chain-enhancer of activated B cells (NF-κB), activator protein 1 (AP-1), and signal transducer and activator of transcription (STAT) pathways, thereby interfering with the signal transduction of pro-inflammatory transcription factors [3]. By curcumin, inhibition of NF-κB may be mediated by the toll-like receptor 4 (TLR-4)-dependent mechanisms [4,5,6]. Moreover, curcumin activates the adenosine monophosphate-activated protein kinase (AMPK), which inhibits a lipid deposition and stimulates glycolysis and fatty acid oxidation in fat and other tissues [7,8]. Recent mouse studies have also implicated curcumin in the recovery from Western-diet-induced intestinal barrier permeability [9,10]. It should be noted that, like other polyphenols, curcumin is poorly absorbed in the intestines and undergoes deep biotransformation by intestinal bacteria [11,12,13,14]. Most of the curcumin detected in the blood of diet-obese mice, which received a curcumin supplement, is found in a glucuronide-conjugated form [12]. Since the relative contribution of curcumin and its metabolites to reported health protective effects has not been evaluated, the molecular mechanisms of these effects require further clarification.

Reports about curcumin’s efficacy against obesity and its associated complications in clinical studies are, to a certain extent, conflicting. While studies show no obesity- and metabolism-related health status alterations or only significant shifts in one of several investigated parameters have been observed, a much wider spectrum of positive outcomes, including improved weight loss, downregulated fat accumulation in adipose tissue and liver, reduced inflammatory cytokines, and oxidative stress markers in blood have been reported [15,16,17,18]. Considering the existing clinical data controversy, developing a universal animal model of obesity for curcumin-related studies with high translational potential may produce a great asset for investigators in the field [19,20]. Different obesogenic diets with varying lipid content have been used in mouse studies to stimulate the development of a high fat diet–induced obesity and its associated pathologic conditions. Most such diets, developed for laboratory mice, provide 45% total calories with fat (high-fat diets; HFD) or 60% total calories with fat (very-high-fat diets; VHFD). A major advantage of VHFD is that they produce obesity in animals faster than HFD and may induce stronger metabolic responses [21,22]. The protocols of curcumin administration also vary substantially between studies. Some investigators administered very low curcumin doses [23,24,25]; others used the PEGylated curcumin derivatives (and not curcumin itself) delivered by a series of intraperitoneal injections [26].

Our study goal was to investigate whether a mouse model of VFHD-induced obesity can be used for evaluating the versatile protective effects of curcumin against a set of manifestations of obesity, which are often investigated separately: insulin resistance, liver pathophysiology, and osteoarthritis. Since most researchers working with mouse models of dietary obesity use mice fed with experimental diets for 4–18 weeks [19], we treated the animals with VHFD for a period of 14 weeks. In our unpublished studies, supplementing the 45%-fat HFD with 0.4% *w*/*w* curcumin resulted in significant improvement of B6 animal metabolic health and immune status. In this study, conducted over the animals housed under identical conditions, we enriched the 60%-fat VHFD with 0.7% *w*/*w* curcumin in order to provide better protection against an increased amount of consumed fat.

## 2. Materials and Methods

### 2.1. Mice and Diets

Five-week-old C57BL/6 male mice, stock No. 000664 (Jackson Laboratory; Bar Harbor, ME, USA) were housed in accordance with National Institutes of Health (NIH) guidelines for the use of experimental animals and the Texas Tech University Institutional Animal Care and Use Committee (IACUC) approval (protocol 16011-04). Animals were kept in individual cages at 23 °C. Upon arrival, they were acclimated in the animal facility for one week. After the acclimation, the animals were randomized and combined in two groups consisting of 10 animals each. The animals of both groups had similar original weight. For 13 weeks, they were fed with a VHFD containing 60% kcal fat, 20% kcal carbohydrate, and 20% kcal protein (Research Diets, Inc.; D12492, New Brunswick, NJ, USA) or VHFD mixed with 0.7% *w*/*w* curcumin powder, obtained from the same provider. The curcumin powder was purchased from Santa Cruz Biotechnology (Santa Cruz, CA, USA). Body composition was measured at week 12 of dietary intervention by using an EchoMRI 3 in 1 Whole Body Composition Analyzer (EchoMRI; Houston, TX, USA). At the end of the dietary interventions, the mice were euthanized by CO_2_ asphyxiation. A cardiac puncture sampled the blood, and serum was separated by using BD microtainers (Becton, Dickinson & Co, Franklin Lakes, NJ, USA). Collected tissue samples (WAT, liver, hind legs) were snap-frozen in liquid nitrogen and placed in storage at −80 °C.

### 2.2. Glucose and Insulin Tolerance Tests (GTT and ITT)

Before the tests, animals were fasted for 5 h. GTT was conducted during week 11 on the diet. The mice were challenged with 2 g/kg body weight d-glucose (Sigma-Aldrich, St. Louis, MO, USA), administered as an intraperitoneal injection of 20% water solution. ITT was conducted during week 13 on the diet. The mice were challenged with 0.75 U/kg body weight human insulin (Humulin, Eli Lilly and Co, Indianapolis, IN, USA), administered as an intraperitoneal injection. For both tests, blood glucose measures were taken before the challenge at 15, 30, 60, 90, and 120 min after administration using an Alpha Track glucometer (Abbott Laboratories, Chicago, IL, USA).

### 2.3. Triglycerides

Quantification of triglycerides in liver and serum used the Triglyceride Colorimetric Assay Kit (Cayman Chemical, Ann Arbor, MI, USA). Collected tissue samples were stored frozen at −80 °C. For the colorimetric analysis, the samples were processed according to the manufacturer’s recommendations. Here, 2-mL liver tissue homogenates were produced from pre-weighed fragments of frozen samples (350–400 mg). Triglyceride concentration in the serum was measured without diluting the samples, and the liver homogenates were diluted five-fold. Absorbance was measured by using a Cytation 3 Cell Imaging Multi-Mode Plate Reader (Biotek, Winooski, VT, USA).

### 2.4. Serum Curcumin and Curcumin Glucuronide Measurements

Quantification of curcumin level and its major metabolite curcumin-β-d-glucuronide in the serum was conducted using liquid chromatography mass spectrometry (LC-MS/MS). For the calibration curve, a 10 μL aliquot of the standard solutions of curcumin and curcumin-β-d-glucuronide and 10 μL hesperetin (internal standard, 10 μg/mL) were added into 70 μL plasma to a final concentration of 5, 10, 50, 200, 500, and 1000 ng/mL curcumin and curcumin-β-d-glucuronide, respectively. The extraction of curcumin from the serum was performed as described before [27]. The solution was briefly vortexed and diluted with 900 μL phosphate buffer saline (PBS) and then extracted with ethyl acetate. The collected organic phase was evaporated to dryness, then reconstituted in 100 μL of mobile phase. After centrifugation, a 25 μL aliquot of the supernatant was injected for LC-MS/MS analysis. The chromatographic separation of curcumin, curcumin-β-d-glucuronide, and hesperetin was performed by using a Vanquish high-performance liquid chromatography (UHPLC) system, with an Acquity BEH C8 column (2.1 mm × 100 mm, 1.7 µm, 130 A, Waters, Milford, MA, USA) under a 10 min isocratic gradient (60% solvent B) at a flow rate of 0.2 mL/min by using solvent A (water, 0.1% formic acid) and solvent B (acetonitrile, 0.1% formic acid). The MS analysis was performed by using a Q-Exactive HF (Thermo Fisher Scientific, Waltham, MA, USA) mass spectrometer in the positive ion mode by using the developed parallel reaction monitoring (PRM) method with the full-scan MS (*m*/*z* 50–7500) and tandem mass spectrometry (MS/MS) by HCD using a collision energy of 35%. The obtained calibration curves for curcumin and its metabolite were used for serum samples quantitation.

### 2.5. Gene Expression

Total tissue RNA was isolated by using an RNeasy Plus Mini kit (Qiagen, Hilden, Germany). cDNA was synthesized from 1 µg RNA using a Maxima H Minus cDNA Synthesis Master Mix (ThermoFisher Scientific, Waltham, MA, USA) and diluted five-fold for use in reverse transcriptase polymerase chain reaction (RT-PCR). For the housekeeping gene expression analysis, this diluted cDNA preparation was additionally diluted 100-fold. RT-PCR was conducted by using PowerUp SYBERGreen Mastermix (Applied Biosystems, Foster City, CA, USA) on a QuantaStudio 3 device (Applied Biosystems, Foster City, CA, USA). The relative gene expression was quantified by ∆ΔCt method, used to determine the mRNA expression and the fold changes, on the QuantStudio Design & Analysis cloud software (Thermo Fisher, Waltham, MA, USA). Primers used were designed by using the Oligoarchitech™ Online software purchased from Sigma-Aldrich (St Louis, MO, USA). For the primer sequences, see Table S1.

### 2.6. Immunohistochemical and Immunofluorescent Staining of WAT

Liver and WAT samples, obtained from euthanized animals, were fixed in Z-Fix 10% aqueous buffered zinc formalin solution (Anatech Ltd., Battle Creek, MI, USA) and stored in 70% ethanol. Before hematoxylin and eosin (H&E) staining, 5-μm paraffin-embedded sections were prepared from the samples. Accumulation of collagen fibers in the liver was stained by using Trichrome Stain (Masson) kit and Weigert’s Iron Hematoxylin set (Sigma-Aldrich, St Louis, MO, USA). The average adipocyte area in WAT was calculated by using AdipoGauge (AdipoGauge @Moussa Lab, 2020 version), a JAVA based, in-house developed software [28].

### 2.7. Osteoarthritis (OA)

Right knee joints were collected from the mice upon euthanization, fixed in 4% paraformaldehyde for 48 h, and decalcified in 10% EDTA-Na_2_ for two months at room temperature as previously described [29]. Following tissue dehydration, the samples were embedded in paraffin, and 5-micron sections were cut at the joint space. The sections were stained with either H&E or Safranin O. Digital pictures were then captured at 100 × magnification. Cartilage degradation scoring was primarily based on Safranin O staining. H&E was used for confirmation. Histopathologic analysis of OA was assessed by determining the severity of loss at the joint space with Safranin O staining. Loss was subjectively scored as 0, no loss of cartilage; 1, mild loss of cartilage at the joint space (1–25% loss); 2, moderate (26–50%); and 3, marked (>50%) by a board certified veterinary pathologist, blinded to the treatment groups.

### 2.8. Statistical Analyzes

The experimental data generated for two groups was analyzed with unpaired T test using GraphPad Prism Software 8.2 (GraphPad, San Diego, CA, USA). Differences were considered significant at *p* ≤ 0.05. Data on graphs are presented as mean ± SEM.

## 3. Results

### 3.1. Food Intake and Body Weight

**Figure 1:**
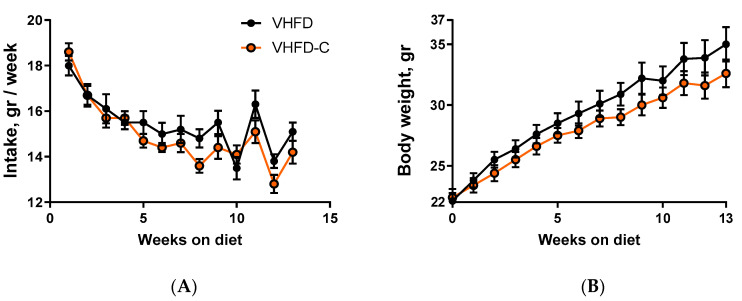
Effect of curcumin on food intake and mouse body weight. Mouse food intake and weight was measured weekly. (**A**) Food intake time course. (**B**) Body weight time course. *p* value for body weight area under the curve is 0.038. *n* = 10.

During 14 weeks of dietary intervention, no significant difference in food intake was observed between the two groups of animals (Figure 1A). Average weekly food consumption during the period of feeding with diet was 15.4 ± 0.4 g for control mice and 13.8 ± 1.2 g for animals fed with curcumin. At the same time, beginning from the first week on the diet and further toward the end of the treatment, the mice that received curcumin supplements gained weight at a slower rate compared to the control group (Figure 1B). By the end of the study, average mouse weight in the control group was 158% compared to their weight at the start of the experiment, and in the curcumin-treated animal group, it was just 144%.

### 3.2. Curcumin Bioavailability

**Figure 2:**
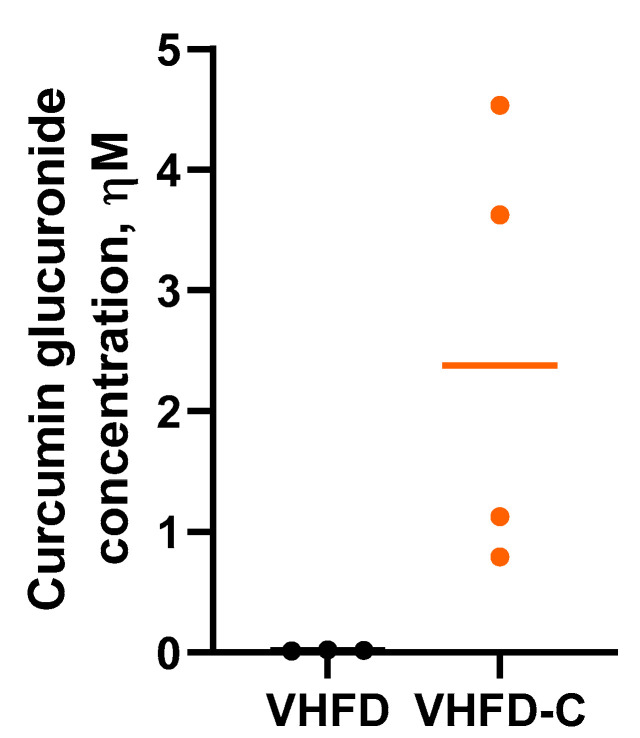
Curcumin glucuronide (CG) bioavailability in mouse serum. Serum was obtained from blood, collected by cardiac puncture during euthanization. CG concentration was determined by liquid chromatography mass spectrometry (LC-MS/MS). *n* = 3–4.

Curcumin and curcumin-β-d glucuronide (principal curcumin metabolite produced by gut bacteria) concentrations were measured in mouse serum. Curcumin itself was not detected in serum collected from curcumin-treated animals, but all these samples contained the curcumin-β-d glucuronide at an average of 2.52 ± 0.92 nM concentration (Figure 2).

### 3.3. Body Adiposity

**Figure 3:**
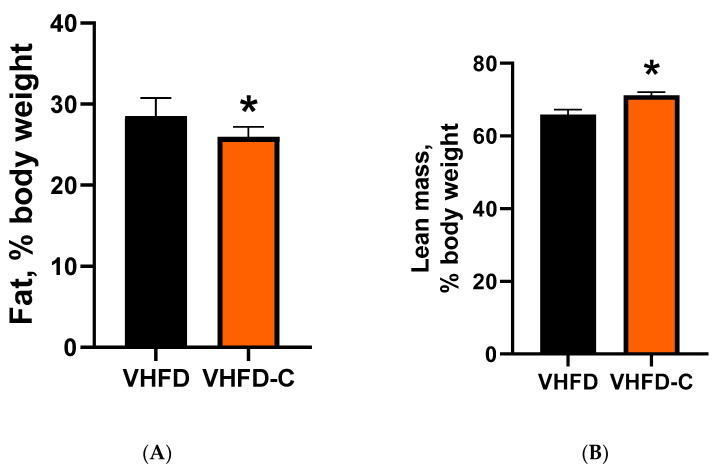
Effect of curcumin on mouse fat and lean body weight. Total body adiposity evaluation was conducted on week 12 of feeding the animals with designated diets on the Whole Body Composition Analyzer (EchoMRI). * indicates a significant difference at *p* value ≤ 0.05. (**A**) Fat body mass. *P* value 0.041. (**B**) Lean body mass. *p* value 0.005. Data represented as mean with standard error of the mean; *n* = 10.

Supplementing the VHFD diet with curcumin reduced the average total body fat content in mice to 25.9% compared to 30.3% in control animals (Figure 3A). Simultaneously, the average lean body weight content in curcumin-treated mice was increased to 70.3% compared to 67.7% in the control group (Figure 3B).

### 3.4. Glucose Metabolism

During the last weeks of dietary intervention, the effects of curcumin on glucose metabolism were evaluated in GTT and ITT. Animals of both groups had similar fasting blood glucose levels, which was confirmed by measurements conducted before challenging them with glucose or insulin, respectively (Figure S2A,C). In the control group, the fasting glucose concentration was between 197 ± 7.9 mg/dL (ITT) and 231 ± 11.1 mg/dL (GTT). In curcumin-treated mice, these levels were between 220 ± 14.0 mg/dL (GTT) and 228 ± 18.2 mg/dL (ITT). The responses to the glucose and insulin challenges also did not reveal differences between the control and curcumin-fed mice (Figure S1A–D respectively).

### 3.5. Adipocyte Cellularity

**Figure 4:**
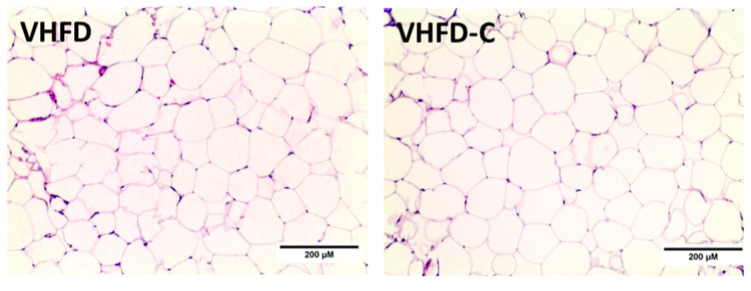
Histologic evaluation of white adipose tissue (WAT). Effect on curcumin on WAT cellularity. Representative images of hematoxylin and eosin (H&E)-stained sections. Magnification 200×; *n* = 4.

Adipocyte hypertrophy is an adaptive adipose tissue response to the excess of energy and correlates positively with metabolic syndrome manifestations [30,31,32,33]. Since, in our study, the curcumin-supplemented diet resulted in body weight and fat mass reduction in mice, we evaluated the effects of curcumin on adipocyte hypertrophy in white epididymal adipose tissue. Hematoxylin and eosin (H&E) staining did not show differences between adipocyte cell size overall between the control and curcumin-treated groups (2873 ± 91 vs. 2738 ± 101 µm^2^, respectively; Figure 4).

### 3.6. Lipid Metabolism and Fibrosis Markers in Liver

**Figure 5:**
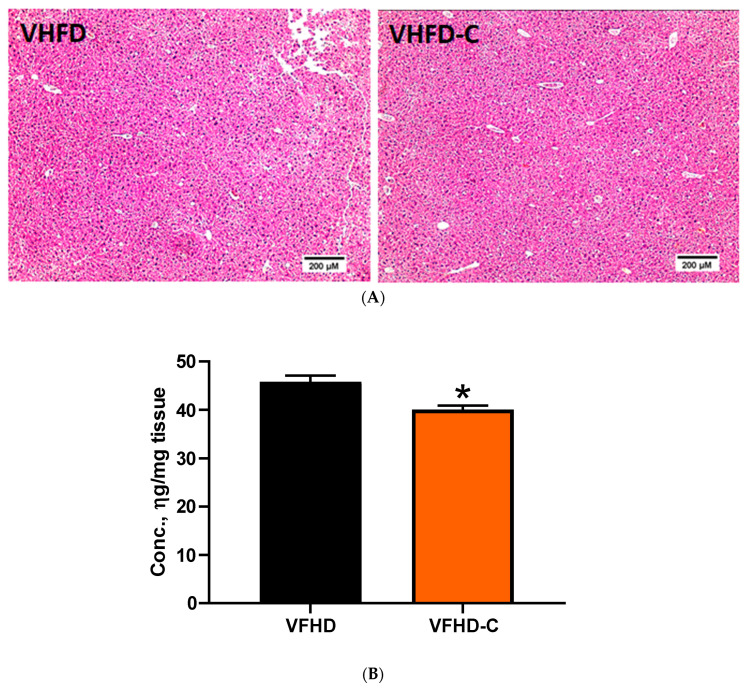
Curcumin effects on liver metabolism in liver. (**A**) Photomicrograph of depicting fat deposition in the liver. Representative images of H&E stained sections. Magnification 100×; *n* = 4. (**B**) Quantification of triglyceride content. Data of colorimetric assay. * indicates a significant difference at *p* value ≤ 0.05. *p* value 0.005; *n* = 9–10.

**Figure 6:**
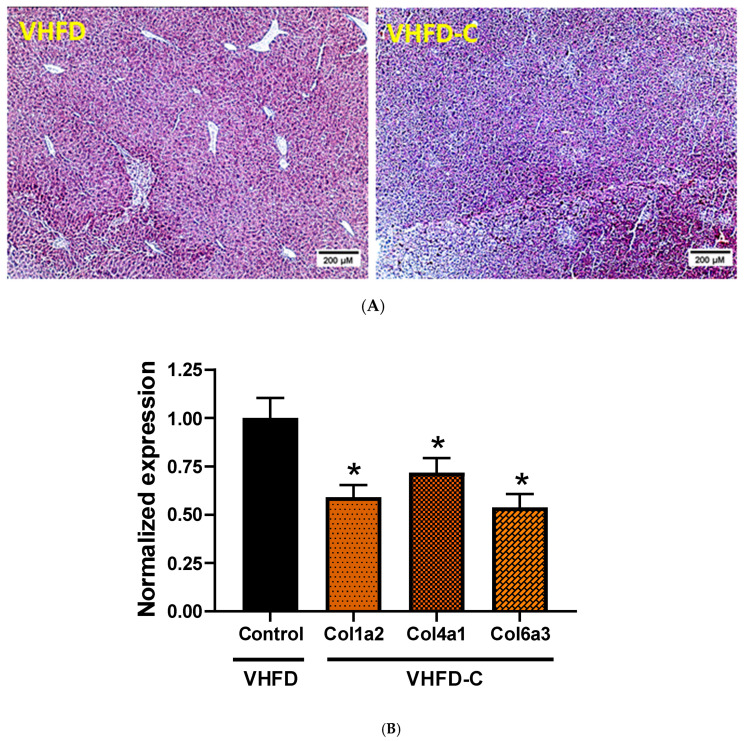
Effects of curcumin on manifestations of fibrosis in mouse liver. (**A**) Photomicrograph of depicting collagen accumulation in the liver. Representative images of Trichrome Stains (Masson) sections. Magnification 100×; *n* = 4. (**B**) Expression of collagen genes in curcumin-fed animals compared to control group. Target gene expression is normalized to TATA box binding protein gene. * indicates a significant difference at *p* value ≤ 0.05. *p* value 0.010, 0.047 and 0.007 respectively; *n* = 5–6.

As feeding mice with VHFD may result in nonalcoholic fatty liver disease (NAFLD), we evaluated the effect of dietary curcumin on fat deposition in the liver. Despite the decrease of liver triglycerides by curcumin by 13% (Figure 5B), H&E staining demonstrated similar fat deposition levels between animals of the two groups (Figure 5A and Figure S2). To ascertain the development of HFD-associated fibrosis in animal liver and whether it may be improved by supplementing mouse diet with curcumin, we evaluated the accumulation of collagen fibers and the expression of fibrosis-related genes. Histopathologic analysis revealed a collagen deposition neither in control nor in curcumin-treated groups of mice (Figure 6A). Expression of three collagen genes, *Col1a2*, *Col4a1,* and *Col6a3*, was downregulated in mice that received curcumin (Figure 6B). Expression of such collagen and pro-inflammatory genes like *Col1a1*, *Col3a1*, *Col5a1*, *Fbn-1*, *ICAM-1*, *MMP-9,* and *TGF-β1* was not different between groups (Figure S3). Expression of *Ctgf*, *MMP-2*, *MMP-13*, *MCP-1*, and *TIMP-1* was below the detection level in both groups.

### 3.7. Knee Joint Pathology Assessment

**Figure 7:**
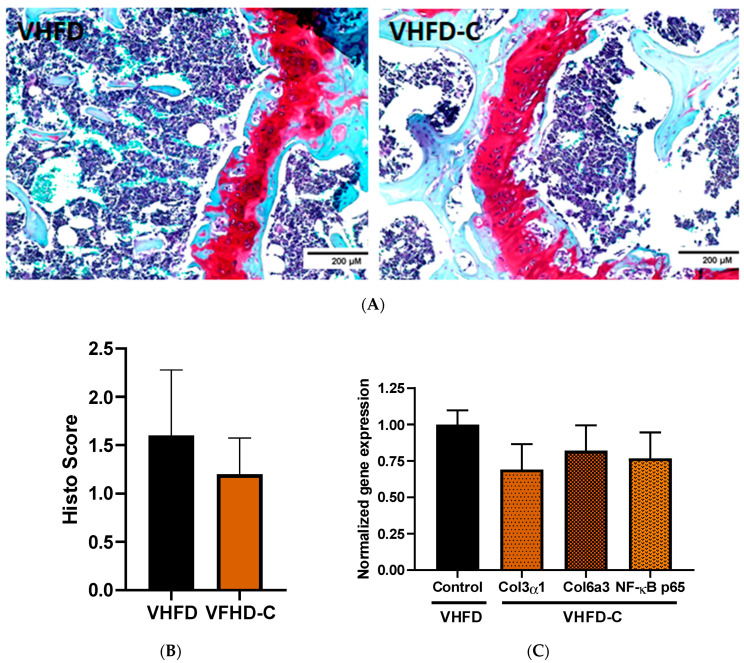
Effects of curcumin on mouse knee cartilage integrity. (**A**) Photomicrograph of mouse joint. Cartilage is shown red on the image. Representative images of Safranin O with the counterstain and H&E stained sections. Cartilage—crimson; collagen—green/blue; nuclei—black. Magnification 200×; *n* = 5. (**B**) Cartilage loss severity score. (**C**) Cartilage gene expression in curcumin-fed animals compared to control group. Target gene expression is normalized to TATA box binding protein gene. *p* value 0.241, 0.490 and 0.381 respectively; *n* = 5–6.

Safranin O staining did not demonstrate significant cartilage tissue degradation of either group (Figure 7A,B). Cartilage loss was not significantly less in the control group than of animals that received the curcumin supplement (Figure 7B). The expression of *Col1α2, Col2α1, Col3α1, Col4α1, Col5α1, Col6α3, Col22α1,* and *NF-κB p65* genes was also similar between control and curcumin-treated mice (Figure 7C and Figure S4). The expression of *Col8α1, Gln3, Gltd8, IL-1β, Pla2g,* and *Ptgs2* inflammation- and OA-related genes was below the detection level.

## 4. Discussion

The anti-obesity effects of curcumin and curcuminoids are being investigated extensively in clinical and animal model-based studies [12,34,35]. In clinical obesity studies, curcumin intake improved a set of pivotal body composition parameters, including reducing body weight, body mass index (BMI), and waist circumference and modulating the adiponectin and leptin levels [34]. Based on the body surface area normalization method of animal-to-human drug dose conversion [36], and daily food consumption rate for mice observed in our previous studies, 0.7% *w*/*w* curcumin corresponds to 5 g/day curcumin consumption by a 75-kg adult human. This dose is consistent with curcumin doses used in clinical trials [2] and 0.01%–1.0% *w*/*w* doses used in published mouse studies using VHFD and curcumin [23,24,25,37]. Consistently with findings from these studies, our curcumin-treated mice demonstrated lower weight gain and percent body fat than the VHFD control group after 14 weeks on diets. On the other hand, the reduced fat content in the curcumin group was not accompanied by reduced adipocyte hypertrophy.

Other studies report rather beneficial morphologic alterations in white adipocyte size in mice fed curcumin. White adipose tissue hypertrophy was reduced significantly in genetically obese *ob/ob* mice fed a regular diet [38]. A trend toward reducing adipocyte size was observed for white fat in a study using C57Bl/6 animals fed a 60% fat VHFD [23]. Of note, in one of these publications, the authors used much higher curcumin dosing compared to our study (3% *w*/*w*), and, in another, the curcumin dosing was much lower (0.08%) than our experiments. Since mechanisms of adipose tissue hypertrophy and hyperplasia still are not fully understood [39,40], the explanation for this phenomenon will require special investigation. It is also worth noting that in some other reports [41], as well as according to our unpublished observations, adding curcumin to HFD and VHFD diets may not affect animal body weight, which suggests that this outcome may be influenced by certain, still unidentified experimental conditions such as composition of the diet or length of feeding different diet-induced obesity models. For instance, Strissel et al. observed cyclic epidydimal fat remodeling dynamics in C57Bl/6 males fed with 60% fat VHFD used in our experiments [42]. In their study, the epidydimal adipocyte volume reached its maximal levels by week 12 of dietary intervention and demonstrated a steep decrease between weeks 12 and 20. It is possible that in our study, VFHD-induced morphologic alterations in control and curcumin-treated animals followed different time courses, and week 14 was not the best time point for demonstrating the effects of curcumin supplements. Fasting blood glucose levels, measured towards the end of dietary intervention, did not differ between the two groups of animals. Glucose and insulin tolerance tests resulted in similar metabolic responses in both groups. Simultaneously, in other mouse studies, curcumin and curcuminoids had a beneficial effect on animal health, observed in animals of different genetic backgrounds, which received their treatment with diet, gavage, or ip injections [37,38,43,44,45,46]. At the same time, curcumin’s effects on glycemia are investigated extensively in rat models [47], and its metabolic outcomes in rats are, to a certain extent, controversial. In rats of different strains, curcumin supplemented with diet or administered by prophylactic gastric gavage, reduced fasting blood glucose and improved insulin sensitivity [48,49]. Nevertheless, similar treatments failed to produce an anti-hyperglycemic effect in other published studies [50,51]. Moreover, curcumin administration in human studies of obese and lean individuals also results in varying outcomes [15,16,17,18,52,53,54]. This suggests that efficacy of curcumin administration against manifestations of obesity-associated carbohydrate metabolism disorders in all kinds of studies is sensitive to the nuances of experimental design. Thus, poorly consistent outcomes of curcumin treatment between published studies make the translational value of curcumin intervention data, generated in animal models of dietary obesity, questionable. Special precautions should be taken when interpreting the VHFD-based data due to the extraordinary high fat content in this type of diet, which does not recapitulate ideally the composition of human obesogenic foods.

Fat-enriched diets providing 32–45% [55,56,57,58] or 60% kcal fat [59,60,61,62,63] are a well-established approach to NAFLD in rodents. However, in our model, evaluating the anti-NAFLD effects of curcumin was problematic. The triglyceride concentration in liver tissue was reduced, but neither visually detectable differences in fat accumulation in the liver nor signs of liver fibrosis were observed in mouse tissues. Interestingly, in studies using “regular” HFD, a period of 9–16 weeks of dietary intervention sufficed to induce NAFLD in mice, but the investigators using 60% fat VHFD treated their mice with the experimental diet for at least 20 weeks [63] and even for 50–80 weeks [59,62]. In the publication, which reports a time course of hepatic steatosis progression in mice fed with VHFD [42], the first signs of disease were observed at week 12 of dietary intervention, with further enhancement of symptoms between week 12 and 20. The modified choline-deficient HFD enriched with methionine produced disease in mice after 14 weeks of treatment [64]. Moreover, in this study, the HFD induced liver fibrosis after 6 weeks in C57BL/6 but not A/J male mice. In Sprague-Dawley rats, VHFD induced hepatic steatosis and inflammation after 16 weeks [60]. All suggests that a more extended period of dietary intervention may be required to develop the robust liver pathology in our model.

OA is a common joint disease associated with chronic pain and disability, bone sclerosis, and degradation of joint tissues [65]. Obesity is one of the principal risk factors of OA etiology [66,67]. Chronic low-grade inflammation is an inalienable co-morbidity of obesity [68,69]. Adipose tissue of obese individuals produces elevated amounts of numerous mediators of inflammation [70,71,72]. Articular cartilage matrix tissue destruction by metalloproteinases, observed in OA, may result from chronic activation of obesity-induced pro-inflammatory intracellular signaling in joints [73,74,75]. A set of clinical studies demonstrated curcumin, curcuminoid, and curcumin-based therapeutics to improve the extremity function and reduce pain and treatment in OA patients [76,77,78,79]. In animal studies using spontaneous and surgical OA models, curcumin treatment protected the chondrocytes against apoptosis and autophagy via activation of Akt/mTOR pathway, reduced collagen accumulation in joints, and inhibited the production of inflammatory mediators [80,81,82]. We evaluated the potential of our model for use in OA-centered mouse studies. However, after 13 weeks on diets, the control mice developed only moderate symptoms of a disease. We did not observe a significant difference in the severity of knee joint cartilage histopathology or expression of OA-associated genes in cartilage tissue between the groups. The VHFD, used in our experiments, was used to induce the disease in several other published OA-focused studies [83,84,85].

Nevertheless, in two of these studies, dietary intervention was not 13 weeks but 20 or 32 weeks [83,84]. Moreover in these papers, as well as in the studies by Griffin et al. and Gu et al. [29,85], the control groups were fed with low-fat diets (LFD), providing 10–13% kcal fat, which facilitated revealing a disease severity difference between groups. In contrast, in our study, the control group of animals was fed with VHFD. Our data suggests that the experimental design used in our study cannot be considered for projects aiming to study the effects of protective bioactives. A comparison against control animals fed with plain obesogenic OA-inducing diets is necessary for evaluating the therapeutic potential of investigated bioactives. Therefore, a time of dietary intervention significantly longer than 13 weeks may be required for OA-centered studies using VFHD to develop the symptoms of robust disease, which may be significantly ameliorated by bioactive food supplements.

## 5. Conclusions

We conclude that, under the experimental conditions used in our study, feeding mice with VHFD for 13 weeks did not result in expected robust manifestations of the targeted pathophysiologic conditions. Our attempt to evaluate the versatile protective effects of curcumin against insulin resistance, liver pathophysiology, and osteoarthritis in VHFD-fed B6 mice encountered essential methodologic limitations. The algorithm for elaborating a model which would develop robust manifestations of metabolism- and immune-status-related pathology in response to 60% kcal fat VHFD requires the optimization of diet composition, length of dietary intervention, and age of animals. Moreover, additional factors not assessed in this paper, must be taken into account (i.e., differences in gut microbiome composition between genetically identical animals housed in different facilities [86]). We recognize that this study produced a limited amount of novel data and that further research is required to unravel the mechanisms underlying the observed pattern of physiologic responses. Nevertheless, we believe that publishing negative data, like those generated in our experiments, may save time and resources for investigators in the field and promote better coordination of efforts in the scientific community.

## Supplementary Materials

The following are available online at https://www.mdpi.com/2218-273X/10/10/1368/s1. Table S1. Primer sequences. Figure S1. Effect of curcumin on glucose metabolism. Figure S2. Curcumin effects on serum triglycerides (TG). Figure S3. Effects of curcumin on gene expression in liver of curcumin-fed animals compared to control group. Figure S4. Effects of curcumin on gene expression in knee cartilage of curcumin-fed animals compared to control group.

## References

[1] Prasad S., Aggarwal B.B., Nd I.F., Benzie F., Wachtel-Galor S. (2011). Turmeric, the Golden Spice: From Traditional Medicine to Modern Medicine. Herbal Medicine: Biomolecular and Clinical Aspects.

[2] Salehi B., Stojanovic-Radic Z., Matejic J., Sharifi-Rad M., Kumar N.V.A., Martins N., Sharifi-Rad J. (2019). The therapeutic potential of curcumin: A review of clinical trials. Eur. J. Med. Chem..

[3] Pulido-Moran M., Moreno-Fernandez J., Ramirez-Tortosa C., Ramirez-Tortosa M. (2016). Curcumin and Health. Molecules.

[4] Long L., Wang J., Chen N., Zheng S., Shi L., Xu Y., Luo C., Deng Y. (2016). Curcumin Ameliorates Reserpine-Induced Gastrointestinal Mucosal Lesions Through Inhibiting IkappaB-alpha/NF-kappaB Pathway and Regulating Expression of Vasoactive Intestinal Peptide and Gastrin in Rats. J. Med. Food..

[5] Ni H., Jin W., Zhu T., Wang J., Yuan B., Jiang J., Liang W., Ma Z. (2015). Curcumin modulates TLR4/NF-kappaB inflammatory signaling pathway following traumatic spinal cord injury in rats. J. Spinal. Cord. Med..

[6] Wang L., Li N., Lin D., Zang Y. (2017). Curcumin protects against hepatic ischemia/reperfusion induced injury through inhibiting TLR4/NF-kappaB pathway. Oncotarget.

[7] Lian N., Jin H., Zhang F., Wu L., Shao J., Lu Y., Zheng S. (2016). Curcumin inhibits aerobic glycolysis in hepatic stellate cells associated with activation of adenosine monophosphate-activated protein kinase. IUBMB Life.

[8] Tong W., Wang Q., Sun D., Suo J. (2016). Curcumin suppresses colon cancer cell invasion via AMPK-induced inhibition of NF-kappaB. uPA activator and MMP9. Oncol. Lett..

[9] Ghosh S.S., Bie J., Wang J., Ghosh S. (2014). Oral supplementation with non-absorbable antibiotics or curcumin attenuates western diet-induced atherosclerosis and glucose intolerance in LDLR-/- Mice—Role of intestinal permeability and macrophage activation. PLoS ONE.

[10] Zhou Y., Zhang T., Wang X., Wei X., Chen Y., Guo L., Zhang J., Wang C. (2015). Curcumin Modulates Macrophage Polarization Through the Inhibition of the Toll-Like Receptor 4 Expression and its Signaling Pathways. Cell Physiol. Biochem..

[11] Lou Y., Zheng J., Hu H., Lee J., Zeng S. (2015). Application of ultra-performance liquid chromatography coupled with quadrupole time-of-flight mass spectrometry to identify curcumin metabolites produced by human intestinal bacteria. J. Chromatogr. B.

[12] Miyazawa T., Nakagawa K., Kim S.H., Thomas M.J., Paul L., Zingg J.M., Dolnikowski G.G., Roberts S.B., Kimura F., Miyazawa T. (2018). Curcumin and piperine supplementation of obese mice under caloric restriction modulates body fat and interleukin-1beta. Nutr. Metab..

[13] Tan S., Rupasinghe T.W., Tull D.L., Boughton B., Oliver C., McSweeny C., Gras S.L., Augustin M.A. (2014). Degradation of curcuminoids by in vitro pure culture fermentation. J. Agric. Food. Chem..

[14] Zam W. (2018). Gut Microbiota as a Prospective Therapeutic Target for Curcumin: A Review of Mutual Influence. J. Nutr. Metab..

[15] Di Pierro F., Bressan A., Ranaldi D., Rapacioli G., Giacomelli L., Bertuccioli A. (2015). Potential role of bioavailable curcumin in weight loss and omental adipose tissue decrease: Preliminary data of a randomized, controlled trial in overweight people with metabolic syndrome. Preliminary study. Eur. Rev. Med. Pharmacol. Sci..

[16] Ganjali S., Sahebkar A., Mahdipour E., Jamialahmadi K., Torabi S., Akhlaghi S., Ferns G., Parizadeh S.M., Ghayour-Mobarhan M. (2014). Investigation of the effects of curcumin on serum cytokines in obese individuals: A randomized controlled trial. Sci. World J..

[17] Rahmani S., Asgary S., Askari G., Keshvari M., Hatamipour M., Feizi A., Sahebkar A. (2016). Treatment of Non-alcoholic Fatty Liver Disease with Curcumin: A Randomized Placebo-controlled Trial. Phytother. Res..

[18] Sahebkar A., Mohammadi A., Atabati A., Rahiman S., Tavallaie S., Iranshahi M., Akhlaghi S., Ferns G.A., Ghayour-Mobarhan M. (2013). Curcuminoids modulate pro-oxidant-antioxidant balance but not the immune response to heat shock protein 27 and oxidized LDL in obese individuals. Phytother. Res..

[19] Fernandes M.R., Lima N.V., Rezende K.S., Santos I.C., Silva I.S., Guimaraes R.C. (2016). Animal models of obesity in rodents. An integrative review. Acta Cir. Bras..

[20] Kleinert M., Clemmensen C., Hofmann S.M., Moore M.C., Renner S., Woods S.C., Huypens P., Beckers J., De Angelis M.H., Schürmann A. (2018). Animal models of obesity and diabetes mellitus. Nat. Rev. Endocrinol..

[21] Hu S., Wang L., Yang D., Li L., Togo J., Wu Y., Liu Q., Li B., Li M., Wang G. (2018). Dietary Fat, but Not Protein or Carbohydrate, Regulates Energy Intake and Causes Adiposity in Mice. Cell Metab..

[22] Takahashi M., Ikemoto S., Ezaki O. (1999). Effect of the Fat/Carbohydrate Ratio in the Diet on Obesity and Oral Glucose Tolerance in C57BL/6J Mice. J. Nutr. Sci. Vitaminol..

[23] Ding L., Li J., Song B., Xiao X., Zhang B., Qi M., Huang W., Yang L., Wang Z.-T. (2016). Curcumin rescues high fat diet-induced obesity and insulin sensitivity in mice through regulating SREBP pathway. Toxicol. Appl. Pharmacol..

[24] Han J.-M., Lee J.-S., Kim H.-G., Seol I.-C., Im H.-J., Cho J.-H., Son C.G. (2015). Synergistic effects of Artemisia iwayomogi and Curcuma longa radix on high-fat diet-induced hyperlipidemia in a mouse model. J. Ethnopharmacol..

[25] Panzhinskiy E., Bashir R., Bagchi D., Nair S. (2019). Effect of Curcumin and α-Lipoic Acid in Attenuating Weight Gain and Adiposity. J. Am. Coll. Nutr..

[26] Liu Y., Cheng F., Luo Y., Zhan Z., Hu P., Ren H., Tang H., Peng M. (2017). PEGylated Curcumin Derivative Attenuates Hepatic Steatosis via CREB/PPAR-gamma/CD36 Pathway. BioMed. Res. Int..

[27] Chen W., Fan-Havard P., Yee L.D., Cao Y., Stoner G.D., Chan K.K., Zhongfa L. (2012). A liquid chromatography-tandem mass spectrometric method for quantification of curcumin-O-glucuronide and curcumin in human plasma. J. Chromatogr. B..

[28] Yosofvand M.L., Kalupahana S., Scoggin N., Moustaid-Moussa S., Moussa N.H. (2020). AdipoGauge software for analysis of biological miscroscopic images. Adipocyte.

[29] Gu H., Li K., Li X., Yu X., Wang W., Ding L., Liu L. (2016). Oral Resveratrol Prevents Osteoarthritis Progression in C57BL/6J Mice Fed a High-Fat Diet. Nutrients.

[30] Albracht-Schulte K., Rosairo S., Ramalingam L., Wijetunge S., Ratnayake C., Kotakadeniya H., Dawson J.A., Kalupahana N.S., Moustaid-Moussa N. (2019). Obesity, adipocyte hypertrophy, fasting glucose, and resistin are potential contributors to nonalcoholic fatty liver disease in South Asian women. Diabetes Metab. Syndr. Obes. Targets Ther..

[31] Arner E., Westermark P.O., Spalding K.L., Britton T., Rydén M., Frisén J., Bernard S., Arner P. (2009). Adipocyte Turnover: Relevance to Human Adipose Tissue Morphology. Diabetes.

[32] Landgraf K., Rockstroh D., Wagner I.V., Weise S., Tauscher R., Schwartze J.T., Löffler D., Bühligen U., Wojan M., Till H. (2014). Evidence of Early Alterations in Adipose Tissue Biology and Function and Its Association With Obesity-Related Inflammation and Insulin Resistance in Children. Diabetes.

[33] Lemieux M., Kalupahana N.S., Scoggin S., Moustaid-Moussa N. (2014). Eicosapentaenoic Acid Reduces Adipocyte Hypertrophy and Inflammation in Diet-Induced Obese Mice in an Adiposity-Independent Manner. J. Nutr..

[34] Akbari M., Lankarani K.B., Tabrizi R., Ghayour-Mobarhan M., Peymani P., Ferns G., Ghaderi A., Asemi Z. (2019). The Effects of Curcumin on Weight Loss among Patients with Metabolic Syndrome and Related Disorders: A Systematic Review and Meta-Analysis of Randomized Controlled Trials. Front. Pharmacol..

[35] Zhao Y., Chen B., Shen J., Wan L., Zhu Y., Yi T., Xiao Z. (2017). The Beneficial Effects of Quercetin, Curcumin, and Resveratrol in Obesity. Oxidative Med. Cell. Longev..

[36] Reagan-Shaw S., Nihal M., Ahmad N. (2007). Dose translation from animal to human studies revisited. FASEB J..

[37] Song Z., Revelo X., Shao W., Tian L., Zeng K., Lei H., Sun H.-S., Woo M., Winer D., Jin T. (2018). Dietary Curcumin Intervention Targets Mouse White Adipose Tissue Inflammation and Brown Adipose Tissue UCP1 Expression. Obesity.

[38] Weisberg S.P., Leibel R., Tortoriello D.V. (2008). Dietary curcumin significantly improves obesity-associated inflammation and diabetes in mouse models of diabesity. Endocrinology.

[39] Choe S.S., Huh J.Y., Hwang I.J., Kim J.I., Kim J.B. (2016). Adipose Tissue Remodeling: Its Role in Energy Metabolism and Metabolic Disorders. Front. Endocrinol..

[40] Haczeyni F., Bell-Anderson K.S., Farrell G.C. (2017). Causes and mechanisms of adipocyte enlargement and adipose expansion. Obes. Rev..

[41] Sarker M.R., Franks S., Sumien N., Thangthaeng N., Filipetto F., Forster M.J. (2015). Curcumin Mimics the Neurocognitive and Anti-Inflammatory Effects of Caloric Restriction in a Mouse Model of Midlife Obesity. PLoS ONE.

[42] Strissel K.J., Stancheva Z., Miyoshi H., Perfield J.W., DeFuria J., Jick Z., Greenberg A.S., Obin M.S. (2007). Adipocyte Death, Adipose Tissue Remodeling, and Obesity Complications. Diabetes.

[43] El-Azab M., Attia F.M., El-Mowafy A.M. (2011). Novel role of curcumin combined with bone marrow transplantation in reversing experimental diabetes: Effects on pancreatic islet regeneration, oxidative stress, and inflammatory cytokines. Eur. J. Pharmacol..

[44] He H.-J., Wang G.-Y., Gao Y., Ling W.-H., Yu Z.-W., Jin T.-R. (2012). Curcumin attenuates Nrf2 signaling defect, oxidative stress in muscle and glucose intolerance in high fat diet-fed mice. World J. Diabetes.

[45] Nishiyama T., Mae T., Kishida H., Tsukagawa M., Mimaki Y., Kuroda M., Sashida Y., Takahashi K., Kawada T., Nakagawa K. (2005). Curcuminoids and Sesquiterpenoids in Turmeric (*Curcuma longa* L.) Suppress an Increase in Blood Glucose Level in Type 2 Diabetic KK-AyMice. J. Agric. Food Chem..

[46] Seo K.-I., Choi M.-S., Jung U.J., Kim H.-J., Yeo J., Jeon S.-M., Lee M.-K. (2008). Effect of curcumin supplementation on blood glucose, plasma insulin, and glucose homeostasis related enzyme activities in diabeticdb/dbmice. Mol. Nutr. Food Res..

[47] Zhang D.-W., Fu M., Gao S.-H., Liu J.-L. (2013). Curcumin and Diabetes: A Systematic Review. Evid.-Based Complement. Altern. Med..

[48] Chougala M.B., Bhaskar J.J., Rajan M., Salimath P.V. (2012). Effect of curcumin and quercetin on lysosomal enzyme activities in streptozotocin-induced diabetic rats. Clin. Nutr..

[49] El-Moselhy M.A., Taye A., Sharkawi S.S., El-Sisi S.F., Ahmed A.F. (2011). The antihyperglycemic effect of curcumin in high fat diet fed rats. Role of TNF-α and free fatty acids. Food Chem. Toxicol..

[50] Majithiya J.B., Balaraman R. (2005). Time-Dependent Changes in Antioxidant Enzymes and Vascular Reactivity of Aorta in Streptozotocin-Induced Diabetic Rats Treated with Curcumin. J. Cardiovasc. Pharmacol..

[51] Nishizono S., Hayami T., Ikeda I., Imaizumi K. (2000). Protection against the diabetogenic effect of feeding tert-butylhydroquinone to rats prior to the administration of streptozotocin. Biosci. Biotechnol. Biochem..

[52] Nieman D.C., Cialdella-Kam L., Knab A.M., Shanely R.A. (2012). Influence of Red Pepper Spice and Turmeric on Inflammation and Oxidative Stress Biomarkers in Overweight Females: A Metabolomics Approach. Plant Foods Hum. Nutr..

[53] Thota R.N., Dias C.B., Abbott K.A., Acharya S.H., Garg M.L. (2018). Curcumin alleviates postprandial glycaemic response in healthy subjects: A cross-over, randomized controlled study. Sci. Rep..

[54] Wickenberg J., Ingemansson S.L., Hlebowicz J. (2010). Effects of *Curcuma longa* (turmeric) on postprandial plasma glucose and insulin in healthy subjects. Nutr. J..

[55] Clapper J.R., Hendricks M.D., Gu G., Wittmer C., Dolman C.S., Herich J., Athanacio J., Villescaz C., Ghosh S.S., Heilig J.S. (2013). Diet-induced mouse model of fatty liver disease and nonalcoholic steatohepatitis reflecting clinical disease progression and methods of assessment. Am. J. Physiol. Liver Physiol..

[56] Li X., Wang Z., Klaunig J.E. (2019). The effects of perfluorooctanoate on high fat diet induced non-alcoholic fatty liver disease in mice. Toxicology.

[57] Melis M., Tang X.-H., Trasino S.E., Patel V.M., Stummer D.J., Jessurun J., Gudas L.J. (2019). Effects of AM80 compared to AC261066 in a high fat diet mouse model of liver disease. PLoS ONE.

[58] Ohashi T., Nakade Y., Ibusuki M., Kitano R., Yamauchi T., Kimoto S., Inoue T., Kobayashi Y., Sumida Y., Ito K. (2019). Conophylline inhibits high fat diet-induced non-alcoholic fatty liver disease in mice. PLoS ONE.

[59] Ito M., Suzuki J., Tsujioka S., Sasaki M., Gomori A., Shirakura T., Hirose H., Ito M., Ishihara A., Iwaasa H. (2007). Longitudinal analysis of murine steatohepatitis model induced by chronic exposure to high-fat diet. Hepatol. Res..

[60] Jensen V.S., Hvid H., Damgaard J., Nygaard H., Ingvorsen C., Wulff E.M., Lykkesfeldt J., Fledelius C. (2018). Dietary fat stimulates development of NAFLD more potently than dietary fructose in Sprague–Dawley rats. Diabetol. Metab. Syndr..

[61] Kim S.W., Hur W., Li T.Z., Lee Y.K., Choi J.E., Hong S.W., Lyoo K.-S., You C.R., Jung E.S., Jung C.K. (2014). Oleuropein prevents the progression of steatohepatitis to hepatic fibrosis induced by a high-fat diet in mice. Exp. Mol. Med..

[62] Velázquez K.T., Enos R.T., E Bader J., Sougiannis A.T., Carson M.S., Chatzistamou I., Carson J.A., Nagarkatti P.S., Nagarkatti M., Murphy E. (2019). Prolonged high-fat-diet feeding promotes non-alcoholic fatty liver disease and alters gut microbiota in mice. World J. Hepatol..

[63] Wu B., Xiao Z., Zhang W., Chen H., Liu H., Pan J., Cai X., Liang G., Zhou B., Shan X. (2019). A novel resveratrol-curcumin hybrid, a19, attenuates high fat diet-induced nonalcoholic fatty liver disease. Biomed. Pharmacother..

[64] Matsumoto M., Hada N., Sakamaki Y., Uno A., Shiga T., Tanaka C., Ito T., Katsume A., Sudoh M. (2013). An improved mouse model that rapidly develops fibrosis in non-alcoholic steatohepatitis. Int. J. Exp. Pathol..

[65] Felson D.T., Anderson J.J., Naimark A., Walker A.M., Meenan R.F. (1988). Obesity and Knee Osteoarthritis. Ann. Intern. Med..

[66] Thijssen E., Van Caam A., Van Der Kraan P.M. (2014). Obesity and osteoarthritis, more than just wear and tear: Pivotal roles for inflamed adipose tissue and dyslipidaemia in obesity-induced osteoarthritis. Rheumatology.

[67] Urban H., Little C.B. (2018). The role of fat and inflammation in the pathogenesis and management of osteoarthritis. Rheumatology.

[68] Liu R., Nikolajczyk B.S. (2019). Tissue Immune Cells Fuel Obesity-Associated Inflammation in Adipose Tissue and Beyond. Front. Immunol..

[69] Mittwede P.N., Clemmer J.S., Bergin P.F., Xiang L. (2016). Obesity and Critical Illness: Insights from Animal Models. Shock.

[70] Burhans M.S., Hagman D.K., Kuzma J.N., Schmidt K.A., Kratz M. (2018). Contribution of Adipose Tissue Inflammation to the Development of Type 2 Diabetes Mellitus. Compr. Physiol..

[71] Engin A. (2017). The Pathogenesis of Obesity-Associated Adipose Tissue Inflammation. Adv. Exp. Med. Biol..

[72] Stolarczyk E. (2017). Adipose tissue inflammation in obesity: A metabolic or immune response?. Curr. Opin. Pharmacol..

[73] Ahmed A.S., Gedin P., Hugo A., Bakalkin G., Kanar A., Hart D.A., Druid H., Svensson C., Kosek E. (2018). Activation of NF-kappaB in Synovium versus Cartilage from Patients with Advanced Knee Osteoarthritis: A Potential Contributor to Inflammatory Aspects of Disease Progression. J. Immunol..

[74] Choi M.-C., Jo J., Park J., Kang H.-K., Park Y. (2019). NF-κB Signaling Pathways in Osteoarthritic Cartilage Destruction. Cells.

[75] Li Y., Jiang W., Wang H., Deng Z., Zeng C., Tu M., Li L., Xiao W., Gao S., Luo W. (2016). Osteopontin Promotes Expression of Matrix Metalloproteinase 13 through NF-kappaB Signaling in Osteoarthritis. BioMed. Res. Int..

[76] Belcaro G., Cesarone M.R., Dugall M., Pellegrini L., Ledda A., Grossi M.G., Togni S., Appendino G. (2010). Product-evaluation registry of Meriva^®^, a curcumin-phosphatidylcholine complex, for the complementary management of osteoarthritis. Panminerva Med..

[77] Nakagawa Y., Mukai S., Yamada S., Matsuoka M., Tarumi E., Hashimoto T., Tamura C., Imaizumi A., Nishihira J., Nakamura T. (2014). Short-term effects of highly-bioavailable curcumin for treating knee osteoarthritis: A randomized, double-blind, placebo-controlled prospective study. J. Orthop. Sci..

[78] Panahi Y., Rahimnia A.-R., Sharafi M., Alishiri G., Saburi A., Sahebkar A. (2014). Curcuminoid Treatment for Knee Osteoarthritis: A Randomized Double-Blind Placebo-Controlled Trial. Phytother. Res..

[79] Pinsornsak P., Niempoog S. (2012). The efficacy of Curcuma Longa L. extract as an adjuvant therapy in primary knee osteoarthritis: A randomized control trial. J. Med. Assoc..

[80] Li X., Feng K., Li J., Yu D., Fan Q., Tang T., Yao X., Wang X. (2017). Curcumin Inhibits Apoptosis of Chondrocytes through Activation ERK1/2 Signaling Pathways Induced Autophagy. Nutrients.

[81] Zhang G., Cao J., Yang E., Liang B., Ding J., Liang J., Xu J. (2018). Curcumin improves age-related and surgically induced osteoarthritis by promoting autophagy in mice. Biosci. Rep..

[82] Zhang Z., Leong D.J., Xu L., He Z., Wang A., Navati M., Kim S.J., Hirsh D.M., Hardin J.A., Cobelli N.J. (2016). Curcumin slows osteoarthritis progression and relieves osteoarthritis-associated pain symptoms in a post-traumatic osteoarthritis mouse model. Arthritis Res..

[83] Barboza E., Hudson J., Chang W.-P., Kovats S., Towner R.A., Silasi-Mansat R., Lupu F., Kent C., Griffin T.M. (2017). Profibrotic Infrapatellar Fat Pad Remodeling without M1 Macrophage Polarization Precedes Knee Osteoarthritis in Mice with Diet-Induced Obesity. Arthritis Rheumatol..

[84] Donovan E.L., Lopes E.B.P., Batushansky A., Kinter M., Griffin T.M. (2018). Independent effects of dietary fat and sucrose content on chondrocyte metabolism and osteoarthritis pathology in mice. Dis. Model. Mech..

[85] Griffin T.M., Huebner J.L., Kraus V.B., Yan Z., Guilak F. (2012). Induction of osteoarthritis and metabolic inflammation by a very high-fat diet in mice: Effects of short-term exercise. Arthritis Rheum..

[86] Webb C.R., Bakker H.D., Koboziev I., Jones-Hall Y., Kottapalli K.R., Ostanin D., Furr K.L., Mu Q., Luo X.M., Grisham M.B. (2018). Differential Susceptibility to T Cell-Induced Colitis in Mice: Role of the Intestinal Microbiota. Inflamm. Bowel Dis..

