# Supplementary material for: Effects of Curcumin in a Mouse Model of Very High Fat Diet-Induced Obesity
Source: Biomolecules. 2020 Sep 25;10(10):1368. doi: 10.3390/biom10101368 (PMC7650718; doi:10.3390/biom10101368)

Supplementary Materials: The following are available online at www.mdpi.com/xxx/s1

Table S1. Primer sequences

Gene	Sequence
18s	F: 5′-GGACAGGATTGACAGATTGATAGC-3′
	R: 5′-TGCCAGAGTCTCGTTCGTTA-3′
TBP	F: 5′-GCCTTCCACCTTATGCTCAG-3′
	R: 5′-GTTGTTGCTGCTGCTGTTG-3′
Arg1	F: 5′-CCTATCACCGCAGAACCT-3′
	R: 5′-GCATCATACAACGAGCAGTG-3′
CD38	F: 5′-TTGCAAGGGTTCTTGAAAC-3′
	R: 5′-CGCTGCCTCATCTACACTCA-3′
CD80	F: 5′-GTCGTCGTCATCGTTGTCAT-3′
	R: 5′-CCGAAGGTAAGGCTGTTGTT-3′
CD206	F: 5′-CCTGTATGCCTGTGATTCG-3′
	R: 5′-GCTTGATATTCTTCTCTTGTCGT-3′
Colα(I)	F: 5′-CCTCAGGGTATTGCTGGACAAC-3′
	R: 5′ACCACTTGATCCAGAAGGACCTT--3′
Col1α2	F: 5′-CCCAGAGTGGAACAGCGATT-3′
	R: 5′-ATGAGTTCTTCGCTGGGGTG-3′
Col3α1	F: 5′-CTGGCCCTCCTGGTGCTTCT-3′
	R: 5′-CCTTGGCCCATCCTTTCCTG-3′
Col4α1	F: 5′-CCTCTTCTCCCTCCCATTGT-3′
	R: 5′-AGCGGGGTGTGTTAGTTACG-3′
Col5α1	F: 5′-AGATTACGAAGTTCCTCAGCGCA-3′
	R: 5′-ATCATCCAGATTCCGGGAGCCAAA-3′
Col6α3	F: 5′-GAGCATCCAGTTCAACCATCAA-3′
	R: 5′-CCTCCACAACCTCCATACCA-3′
Col8α1	F: 5′-TCTGCCACCTCAAATCCCTCCTCA-3′
	R: 5′-TCTCCGCGCAAACTGGCTAACG-3′
CTGF	F: 5′-ACTGCCCCTTCCCGAGAA-3′
	R: 5′-TCCTTGGGCTCGTCACACA-3′
FASN	F: 5′-TGTATCCTGTGTCCAACCT-3′
	R: 5′-GGCTTGTCCTGCTCTAACTG-3′
Fn1	F: 5′-AGAACCAGAGGAGGCACAAG-3′
	R: 5′-CCGTGTAAGGGTCAAAGCAT-3′
Gln3	F: 5′-GGATATTCAAGTTGGAGT-3′

	R: 5′-GAGTTACAAGGTGAGATAA-3′
Gltd8	F: 5′-ACATTGGTTATCTTGACTA-3′
	R: 5′-AGTTCTGCTGTATAATCC-3′
ICAM-1	F: 5′-CCGCTGTGCTTTGAGAACT-3′
	R: 5′-GGTCCTTGCCTACTTGCTG-3′
IL-1β	F: 5′-GTCACAAGAAACCATGGCACAT-3′
	R: 5′-GCCCATCAGAGGCAAGGA-3′
IL-10	F: 5′-GGTTGCCAAGCCTTATCG-3′
	R: 5′-TCCACTGCCTTGCTCTTAT-3′
MCP-1	F: 5′-ACTTCTATGCCTCCTGCTCAT-3′
	R: 5′-CCTGCTTGTGATTCTCCTGTAG-3′
MMP2	F: 5′-GATACCCTCAAGAAGATGCAGAAGTT-3′
	R: 5′-ACATCTTGGCTTCCGCATG-3′
MMP9	F: 5′-GAAGGCAAACCCTGTGTGTT-3′
	R: 5′-AGAGTACTGCTTGCCCAGGA-3′
MMP13	F: 5′-ACTTAACTTACAGGATTGTGAACTATACTCCT-3′
	R: 5′-TGTCAGCAGTGCCATCATAGATT-3′
NF-kB p65	F: 5′-GAAGCACAGATACCACCAAGAC-3′
	R: 5′-TCAGCCTCATAGTAGCCATCC-3′
Pla2g	F: 5′-GCTCCTGAACCTGAACAAGATG-3′
	R: 5′-TGCCACCAAGTCCACAGT-3′
Ptgs2	F: 5′-GCATTCATTCCTCTACAT-3′
	R: 5′-CAACACTCACATATTCATAC-3′
STAT1	F: 5′-GACTTCAGACACAGAAATCAACTC-3′
	R: 5′-TTGACAAAGACCACGCCTT-3′
TGFβ1	F: 5′-GCCCGAAGCGGACTACTATG-3′
	R: 5′-AGATGGCGTTGTTGCGGT-3′
TLR4	F: 5′-AGTAGCACTGACACCTTCCTT-3′
	R: 5′-GCCTTAGCCTCTTCTCCTTCA -3′
TIMP1	F: 5′-CATGGAAAGCCTCTGTGGATATG-3′
	R: 5′-GATGTGCAAATTTCCGTTCCTT-3′

Figure S1. Effect of curcumin on glucose metabolism. Before each test, the animals were fasted for 5 hours. Blood glucose levels were measured at 15, 30, 60, 90, and 120 min after administration of glucose or insulin. A) GTT: blood glucose levels time course. Test was conducted on week 11 of feeding with diets. Mice were challenged with 2 g/kg body weight glucose. B) GTT: area under the curve. C) ITT: blood glucose levels time course. Test was conducted on week 13 of feeding with diets. Mice were challenged with 0.75 U/kg body weight human insulin. D) ITT: area under the curve. Quantification of metabolic responses during the tests was performed by comparing the area under the curve. Data represented as mean with standard error of the mean; n = 7-10.

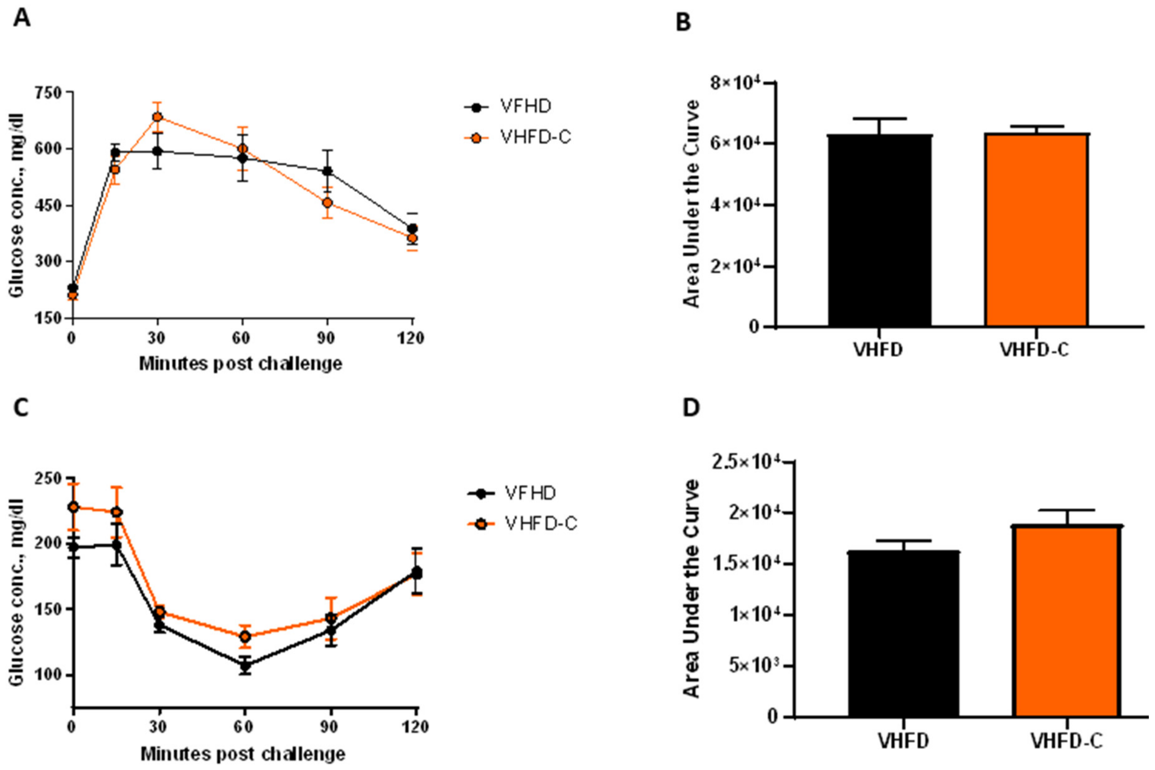

Figure S2. Curcumin effects on serum triglycerides (TG). Data of colorimetric assay. N = 10.

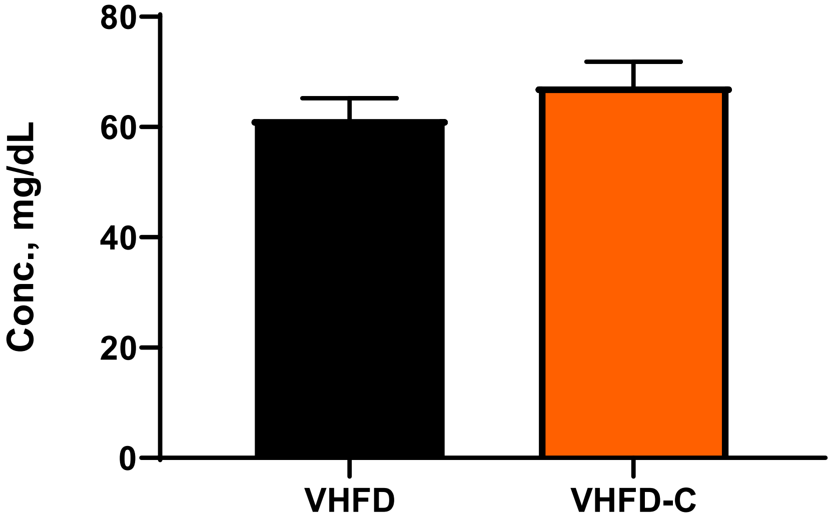

Figure S3. Effects of curcumin on gene expression in liver of curcumin-fed animals compared to control group. Target gene expression is normalized to TATA box binding protein gene. N = 5-6.

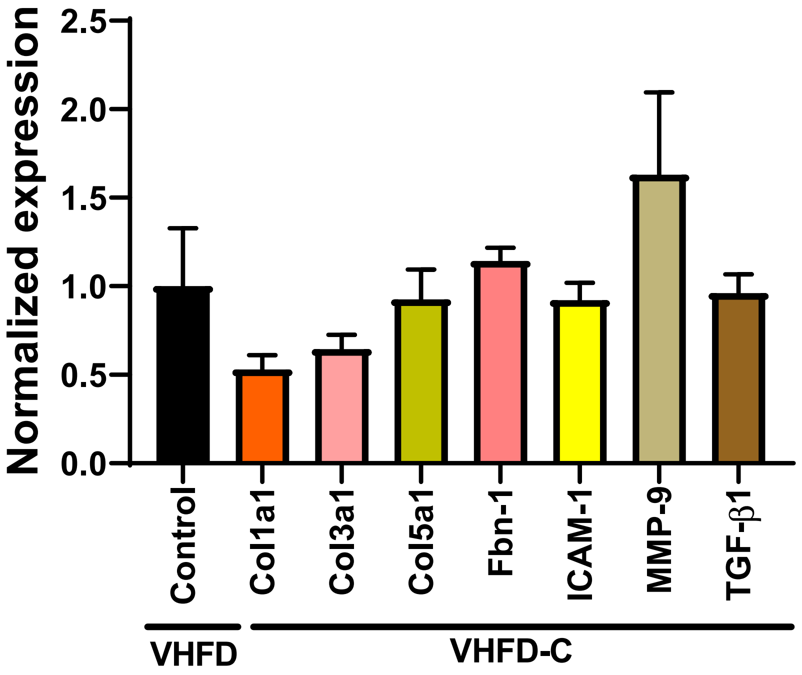

Figure S4. Effects of curcumin on gene expression in knee cartilage of curcumin-fed animals compared to control group. Target gene expression is normalized to TATA box binding protein gene. N = 6-7.

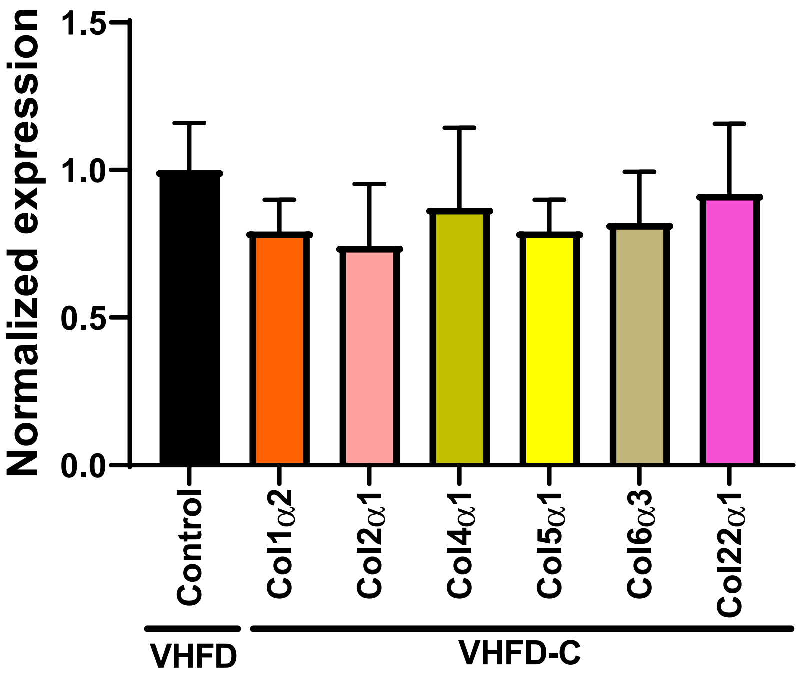

Supplement: Supplementary file 1 [file biomolecules-10-01368-s001.pdf]
